# Molecular epidemiology and clinical characteristics of respiratory syncytial virus in hospitalized children during winter 2021–2022 in Bengbu, China

**DOI:** 10.3389/fpubh.2023.1310293

**Published:** 2024-01-03

**Authors:** Limin Huang, Yuanyou Xu, Yanqing Yang, Hongming Dong, Qin Luo, Zhen Chen, Haijun Du, Guoyong Mei, Xinyue Wang, Yake Guan, Chihong Zhao, Jun Han, Guoyu Lu

**Affiliations:** ^1^The First Affiliated Hospital of Bengbu Medical College, Bengbu, Anhui, China; ^2^Jinling Hospital, Affiliated Hospital of Medical School, Nanjing University, Nanjing, China; ^3^Chinese Center for Disease Control and Prevention, Beijing, China; ^4^School of Basic Medicine, North China University of Science and Technology, Tangshan, China; ^5^National Key Laboratory of Intelligent Tracking and Forecasting for Infectious Diseases, National Institute for Viral Disease Control and Prevention, Chinese Center for Disease Control and Prevention, Beijing, China; ^6^College of Life Science and Agriculture and Forestry, Qiqihar University, Qiqihar, China

**Keywords:** respiratory syncytial virus, molecular epidemiology, clinical characteristics, G gene, amino acid mutation

## Abstract

**Objective:**

This study aimed to study the molecular epidemiology and clinical characteristics of respiratory syncytial virus (RSV) infection from hospitalized children with ARTI in Bengbu.

**Methods:**

One hundred twenty-four nasopharyngeal swab specimens and clinical data from children with ARTI cases were collected in Bengbu, China, during winter 2021–2022. The samples were detected by qPCR of 13 respiratory viruses. Phylogenetic analysis was constructed using MEGA 7.0. All analyses were performed using SAS software, version 9.4.

**Results:**

In winter 2021–2022, URTI, NSCAP, SCAP, and bronchiolitis accounted for 41.03%, 27.35%, 17.09%, and 14.53% of hospitalized children in Bengbu, China. The detection rates of the top three were RSV (41.94%), ADV (5.65%), and FluB (5.65%) in hospitalized children through 13 virus detection. RSV is the main pathogen of hospitalized children under 2 years old. Forty-eight sequences of G protein of RSV were obtained through PCR amplification, including RSV-A 37 strains and RSV-B 11 strains. Phylogenetic analysis showed that all RSV-A and RSV-B were ON1 and BA9 genotypes, respectively. ON1 genotypes were further divided into two clades. The majority of ON1 strains formed a unique genetic clade with T113I, V131D, N178 G, and H258Q mutations. Furthermore, RSV infection was an independent risk factor for ventilator use (OR = 9.55, 95% CI 1.87–48.64).

**Conclusion:**

There was a high incidence of RSV among hospitalized children during winter 2021–2022 in Bengbu with ON1 and BA9 being the dominant strains. This study demonstrated the molecular epidemiological characteristics of RSV in children with respiratory infections in Bengbu, China.

## 1 Introduction

Respiratory syncytial virus (RSV) is an extremely common, airborne RNA virus, mainly affecting infants and the older adult. RSV is the leading pathogen causing acute lower respiratory tract infection (ALRTI) such as bronchiolitis and pneumonia in infants under 6 months old and young children and lower respiratory tract diseases that may endanger lives in children under 5 years old, the infirm, and the older adult (1, 2). Most children are infected with RSV under the age of 2 years (3), with up to 90% of children experiencing RSV-related bronchiolitis during their first few years of life (4). According to the data from the World Health Organization (WHO), approximately 34 million children are infected with RSV each year, of which approximately 66,000 to 199,000 fatalities from RSV infection, which is an important factor leading to child mortality (5). Globally, RSV causes over 336,000 older adult hospitalizations and 14,000 deaths annually (6). It is estimated that up to 2,500 children are hospitalized every day due to RSV infection in China, which is one of the countries with the largest number of children with LRTI caused by RSV in the world (7).

RSV is a negative-sense, single-stranded RNA virus that belongs to the family *Pneumoviridae* and the genus *Orthopneumovirus* (8). The RSV genome contains 10 genes and encodes 11 proteins, including NS1, NS2, N, P, M, SH, G, F, M2-1, M2-2, and L (9). The attachment glycoprotein (G) and fusion glycoprotein (F) are the main target antigens for neutralizing antibodies and vaccine development. RSV is divided into two subtypes, A and B, based on the G protein antigen (10). Based on the variations of the second hypervariable region (HVR2) of G protein, RSV-A is categorized into 22 genotypes and RSV-B is subdivided into 36 genotypes (11, 12). At present, the ON1 and BA9 are the dominant genotypes prevalent globally, including China (13–16).

From RSV surveillance data of 14 countries based on the Global Influenza Surveillance and Response System (GISRS) (17), RSV showed a retaliatory rebound after the COVID-19 epidemic in the autumn and winter of 2022 in Canada and the United States (18). According to reports, the positive detection rate of RSV in Canada was significantly higher in the autumn and winter of 2022 than in previous years (19), and the positive detection rate of RSV in the United States increased significantly in the autumn of 2022, and the epidemic peak is earlier than in previous years (20). During the epidemic season before 2021, the RSV detection rate remained at an extremely low level in Hubei, China, while a moderate epidemic (approximately 10%) occurred in the same period in 2021 (21).

Therefore, to understand the epidemiological features of RSV during the COVID-19 disease epidemic during winter 2021–2022 in Bengbu, Anhui, China, the genetic diversity and molecular evolution of RSV were analyzed in this study. We also analyzed RSV’s impact on respiratory diseases in children, especially pneumonia in children.

## 2 Methods

### 2.1 Study population and specimen collection

One hundred twenty-four nasopharyngeal swab specimens were collected from the enrolled cases with acute respiratory tract infections (ARTI) from the First Affiliated Hospital of Bengbu Medical College in hospitalized children in Bengbu from October 2021 to January 2022. These cases contained upper respiratory tract infection (URTI) and community-acquired pneumonia (CAP). A URTI was defined as fever (body temperature ≥ 38°C) accompanied by respiratory signs or symptoms (i.e., cough, sore throat, and rhinorrhea). CAP was defined in accordance with the guidelines for the management of community-acquired pneumonia in children in China (the revised edition of 20,130) (22). All cases were investigated by clinicians using a uniform questionnaire that included demographic data, epidemiological data, and clinical manifestations.

### 2.2 Identification of respiratory viruses by qPCR

Total viral nucleic acids (RNA and DNA) were extracted from the viral transportation medium using the Fujian Baineng Medical Technology Co., Ltd. Thirteen respiratory viruses were simultaneously detected with real-time PCR, including RSV, influenza virus A (FluA), influenza B (FluB), human coronavirus (NL63, OC43, 229E, and HKU1), parainfluenza virus 1 to 3 (PIV), human metapneumovirus (HMPV), adenovirus (ADV), and human bocavirus (HBoV). The nucleic acids were stored at −80°C until further use.

### 2.3 PCR amplification and G gene sequencing

RSV cDNA was obtained from the extract using MultiScribe reverse transcriptase and random hexamers. Total cDNA was used in a PCR (Century 2 × Es Taq MasterMix (Dye), CWBIO). The amplification follows conditions: 94°C for 2 min, followed by 40 cycles of 94°C for 30s, 56°C for 45 s, 72°C for 30s, and a final extension at 72°C for 5 min. The PCR products were sequenced using an ABI Prism 3730XL DNA Analyzer at Tsingke Co., Ltd. (Beijing, China). The sequences were edited using Sequencher software version 5.0 (Gene Codes, Ann Arbor, MI, United States). These sequences were deposited in GenBank with accession numbers from QQ933800 to QQ933847.

### 2.4 Analysis of phylogenetic and amino acid replacement

SeqMan program (DNASTAR 7.0, Inc., Madison, WI) was used for contigs assembling and obtaining the full length of the G gene. The sequences obtained in this study were aligned with representative sequences retrieved from GenBank using Clustal W. The phylogenetic tree was constructed using the maximum likelihood with HKY+ G and TN93+ G models for RSV-A and RSV-B. The reliability of the tree topology was evaluated by bootstrapping with 1,000 replications in Mega 7.0 software. Deduced amino acid sequences were translated with the standard genetic code using MEGA software version 7.0. The sequences of the RSV-A strains and RSV-B strains were aligned with the prototype strain ON67-1210A and BA4128/99B, respectively. RSV-A and RSV-B sequences were downloaded from the GenBank database, respectively.

### 2.5 Analysis of the N-glycosylation site

Putative N-glycosylation sites were predicted using NetNGlyc 1.0 webserver^1^ to identify the sequence motifs N-X-S/T (sequon), where X can be any amino acid except proline. Only the sites with scores higher than 0.5 were accepted as glycosylated.

### 2.6 Statistical analysis

The clinical data were entered using Epidata 3.0 and organized using Excel 2019. SAS 9.4 was used for the statistical analysis, and continuous variables were presented as median (interquartile range, IQR) and compared with the Kruskal–Wallis test and *t*-test between different groups; categorical variables were presented as number (%) and compared by chi-square test or Fisher’s exact test between different groups. A *p*-value of <0.05 was considered statistically significant. A flow chart about the methodology of sampling and analyses is given in Supplementary Figure S1.

## 3 Results

### 3.1 The epidemiological characteristics of RSV

From October 2021 to January 2022, a total of 124 samples with ARTI were collected from hospitalized pediatric patients in Bengbu, Anhui, China. Of 124 children, 117 cases had complete clinical information. In this study, the majority (36.75%, 43 of 117) of the patients were younger than 6 months, 30.77% (36 of 117) of cases were 6 months to 2 years old, 23.93% (28 of 117) of cases were 2 years old to 5 years old, and 8.55% (10 of 117) of children’s cases were over 5 years old (range from 1 month old to 16 years old). The male-to-female ratio was 1.74:1. Of these samples, RSV infection was the main infection, accounting for 41.94% (52 of 124), followed by ADV 5.65% (7 of 124), FluB 5.65% (7 of 124), HBOV 2.42% (3 of 124) and HCoVs 0.81% (1 of 124). Therefore, subsequent studies focused on analyzing the pathogenic and epidemiological characteristics of RSV.

**Figure 1:**
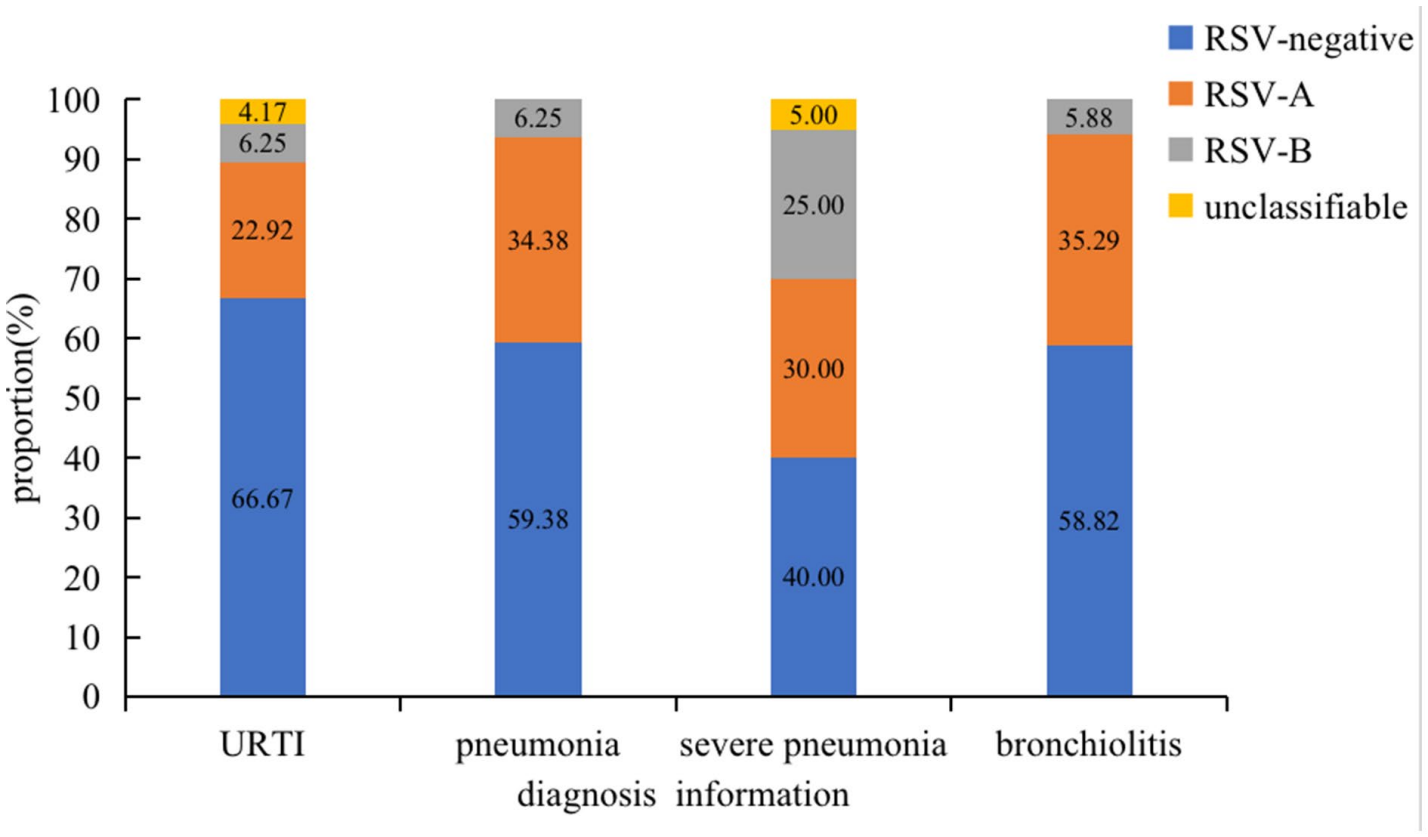
Proportion rates of RSV subtypes of different infection types.

Of the 52 RSV-positive cases, the proportion of RSV-A and RSV-B was 71.15% (37 of 52) and 21.15% (11 of 52), respectively. The other 7.69% (4 of 52) cases were unclassifiable. The median age of the RSV-infected patients was 1 year old (IQR: 0.3–2.5 years old), and RSV infection mainly affected children under 2 years old (79.17%, 38 of 48). Excluding four cases of missing clinical information, the proportion rates of RSV of the four types of cases mainly occurred in SCAP (60%), followed by NSCAP (41.8%), bronchiolitis (40.6%), and URTI (33.3%) (Figure 1). RSV-A was the dominant strain in this study.

### 3.2 Phylogenetic analysis of RSV G

**Figure 2:**
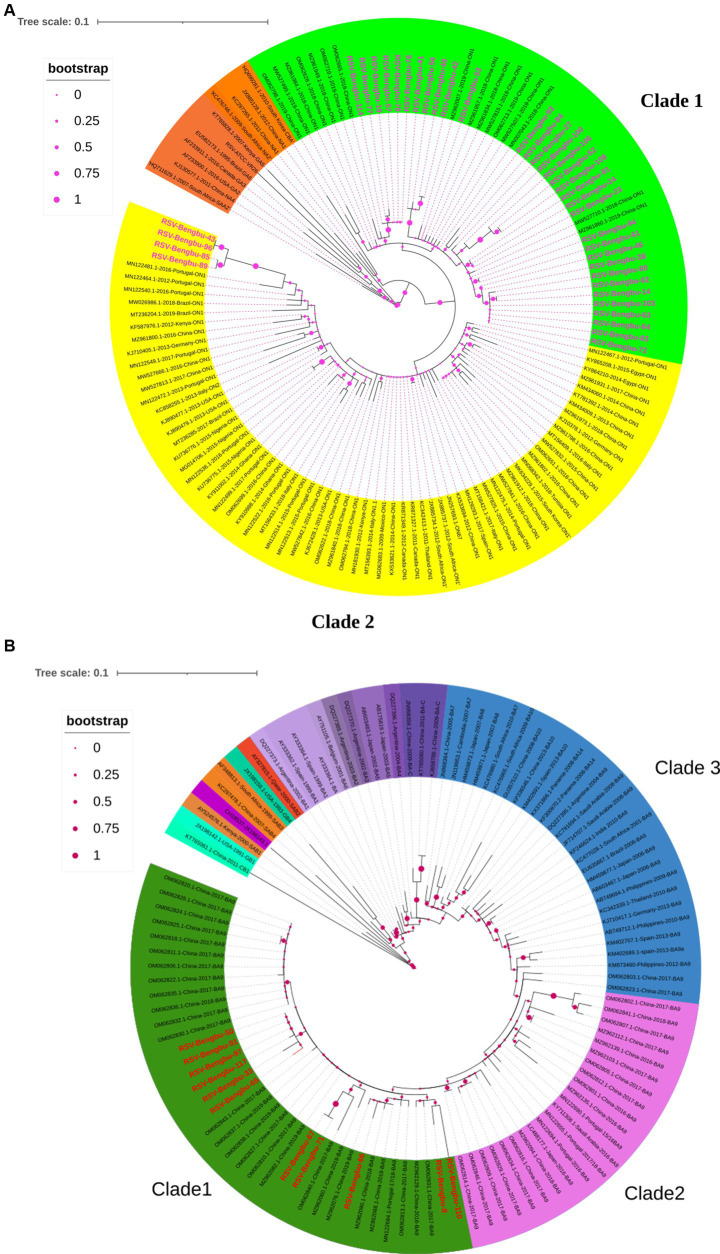
Phylogenetic tree in RSV-A **(A)** and RSV-B **(B)**. The phylogenetic tree of RSV-A and RSV-B G protein genes was constructed by maximum-likelihood method. The reference sequence used to build the tree was downloaded from the GenBank database. **(A)** The phylogenetic tree of RSV-A, with the studied strain labeled pink. ON1 genotype divided into two clades, with green indicating clade 1 and yellow indicating clade 2, and the branch nodes show bootstrap values with pink dots. **(B)** The phylogenetic tree of RSV-B, with the studied strain labeled red. BA9 divided into three clades, with green indicating clade 1, pink indicating clade 2, blue indicating clade 3, and the branch nodes show bootstrap values with peach dots.

In total, 40 full-length G genes (30 RSV-A and 10 RSV-B) and 8 HVR2 sequences of G gene (7 RSV-A and 1 RSV-B) were obtained by PCR amplification for subsequent analysis. Phylogenetic analysis showed that all RSV-A strains and RSV-B strains were of the ON1 and BA9 genotypes, respectively (Figures 2A,B). Thirty-seven ON1 genotype was further divided into two branches in this study. Thirty-three strains were only clustered with reference sequences from China in clade 1, and 4 strains were clustered with reference sequences from Portugal, China, Italy, Brazil, Kenya, the United States, and 13 other countries or regions in clade 2 (Figure 2A). In this study, the BA9 genotype of clade 1 was mainly clustered with the Chinese BA9 genotype sequences (Figure 2B). The nucleotide homology of HVR2 of ON1 and BA9 genotypes was 92.5%–100% and 94.8%–100%, respectively. The calculated overall mean distance was 0.031 for RSV-A and 0.033 for RSV-B.

### 3.3 Amino acid substitution of G glycoprotein

**Figure 3:**
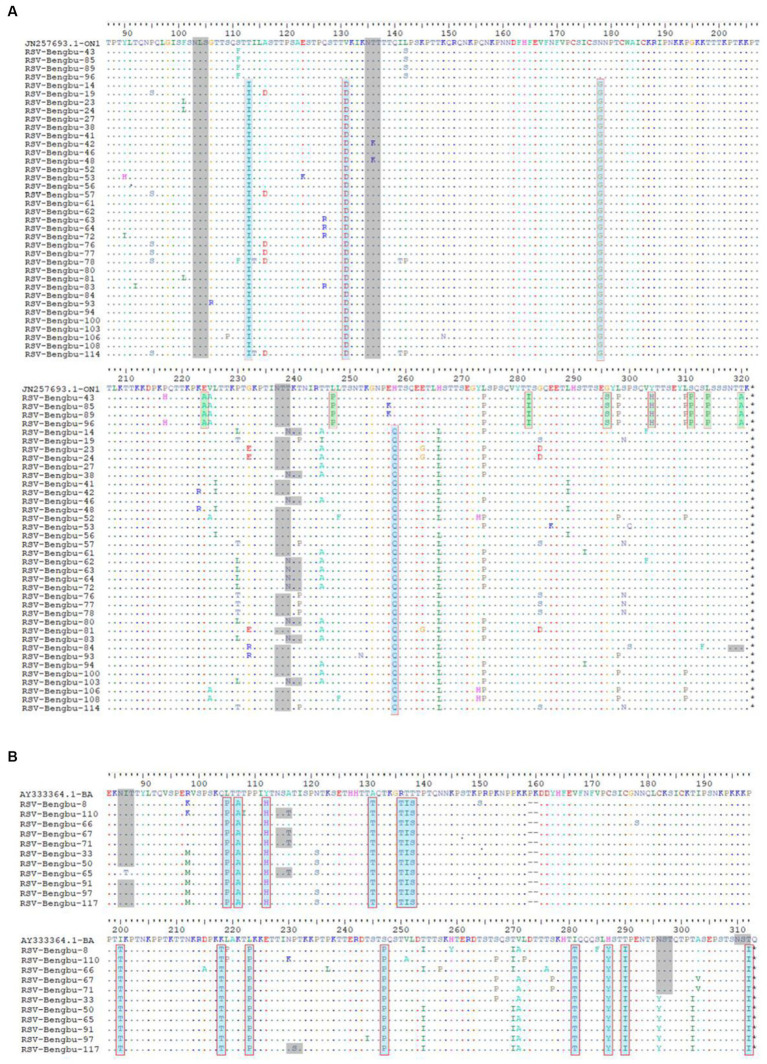
Deduced amino acid sequences alignment of the G protein of RSV sequences. **(A)** Deduced amino acid sequence alignment of the ON1 genotype G protein relative to the prototype strain ON67-1210A. The dots indicate aa identical to ON67-1210A; the major mutations of clade 1 are shown in cyan, while those of clade 2 are shown in green. The putative N-glycosylation sites are shown in gray shading. **(B)**: The G protein amino acid (aa) partial sequences of BA strains were aligned with the prototype BA4218/99B. The dots indicate aa identical to the prototype BA4218/99B, the major mutations of amino acid are shown in cyan. The putative N-glycosylation sites are shown in gray shading.

**Figure 4:**
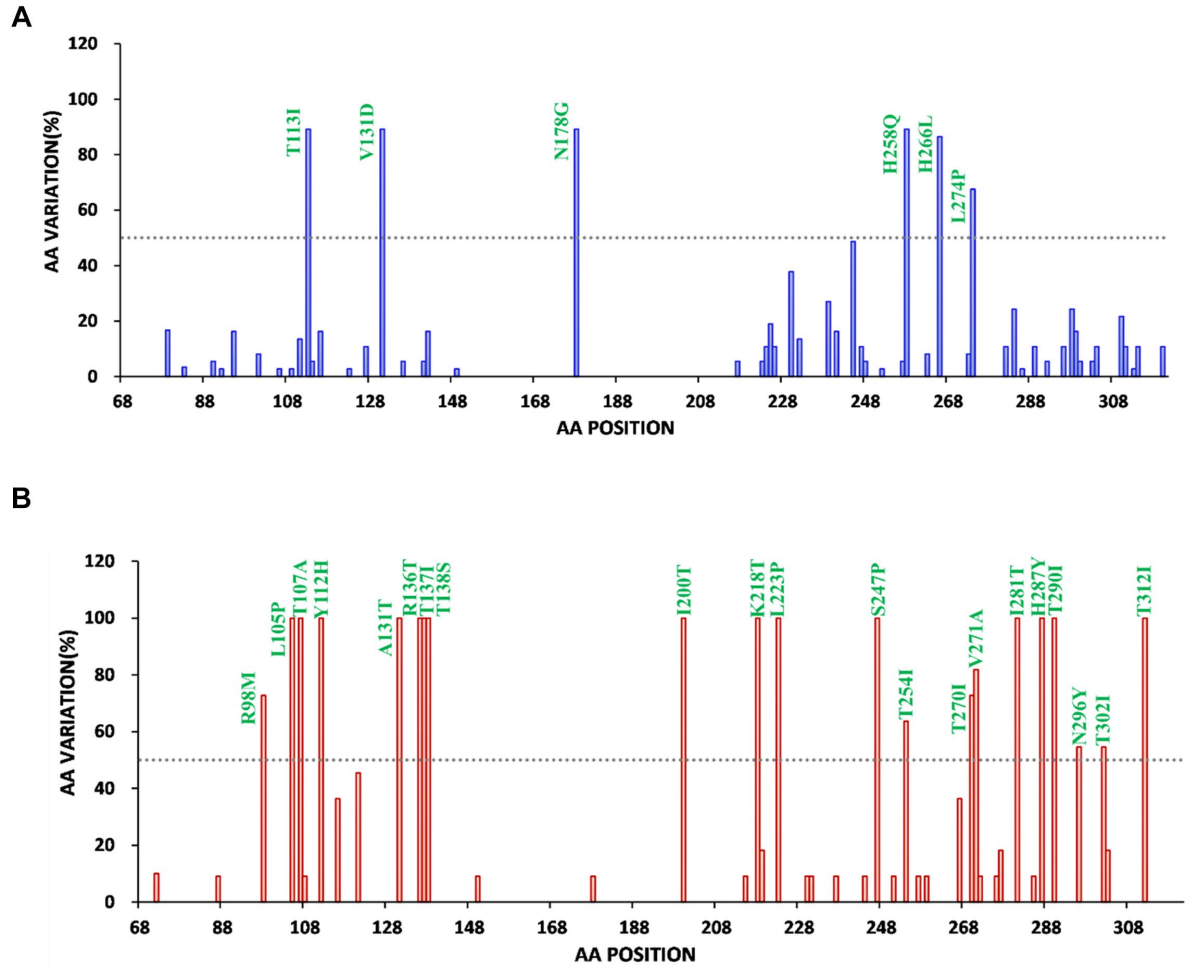
Frequency map of amino acid changes in the extracellular domain of Bengbu RSV G protein of RSV-A **(A)** and RSV-B **(B)** (a, aa 68 to 321.b, aa 68 to 310), **(A)** RSV-A reference strain is ON67-1210 (JN257693) (blue). **(B)** RSV-B reference strain is BA4128/99B (AY333364) (red). Compared with the original strain, the amino acid substitution with a frequency of >50% (dotted line) was marked.

Subsequently, the diversity of amino acid mutations of ON1 and BA9 was analyzed. The most common amino acid substitutions of the G glycoprotein gene of RSV stains were identified in mucin-like regions 1 and 2 compared to the prototype ON1 (JN257693) strains and BA1 strains (AY333364) (Figures 3, 4). The most common substitutions of the G protein gene of ON1 strains were T113I, V131D, N178G, T245A, H258Q, H266L, and L274P in this study (Figure 3A). Additionally, the majority of ON1 strains with T113I, V131D, N178 G, and H258Q mutations formed a unique genetic cluster. N178 G was located near the CX3C motif binding to CX3CR1 to initiate infection. H258Q was observed within the 24aa duplication region of G glycoprotein. Moreover, eight amino acid substitutions occurred in the majority of BA9 strains, including R98M, N121S, T254I, T270I, V271A, N296Y, T302I, and N178S. Similarly, N178S substitution was also found in the BA9 strain (Figures 3B, 4B).

### 3.4 N-glycosylation sites of G protein

To predict N-linked glycosylation sites, amino acid sequences of G protein were submitted to online bioinformatics tools (NetNGlyc). For ON1 genotypes, three major putative N-glycosylation sites, N103, N135, and N237 were identified (Figure 3A, gray shading). N-glycosylation site N239 was also identified in the G gene of 23% ON1. For BA9 genotype, the N-glycosylation site N310, N318, N86 (91%), N114 (36%), and N296 (45%) were also predicted in this study that were similar with BA prototype strain (Figure 3B, gray shading). In addition, one of the BA9 strains carried one N-glycosylation site at aa 230 due to the P231S substitution (Figure 3).

### 3.5 Clinical characteristics of children infected with RSV

To understand the clinical symptoms of children infected with RSV, the differences in clinical symptoms, laboratory, radiographic findings, treatments, and outcomes were analyzed between RSV-infected and non-infected children. The results showed that the most common clinical manifestations in RSV-infected cases were cough (83.33%), pulmonary rales (81.25%), sputum (70.83%), fever (62.5%), and wheezing (60.42%) (Table 1). The median age of RSV-infected children was lower than that of RSV-uninfected children (0.45 years old vs. 1.2 years old *p* < 0.01) (Table 1). Compared to patients without the RSV infection, the RSV-infected patients were more likely to produce sputum (70.83%, 34/48, *p* = 0.030), wheezing (60.42%, 29/48, *p* < 0.01), pulmonary rales (81.25%, 39/48, *p* < 0.01), and chest shadow (26.32%, 10/38, *p* = 0.048) (Tables 1, 2). In addition, patients with RSV infection were more likely to require mechanical ventilation (35.42%, 17 of 48, *p* < 0.01) (Table 2), in which children infected with RSV-B need more mechanical ventilation than those with RSV-A (63.64% *VS* 26.47%, *p* = 0.035) (Supplementary Tables S1, S2).

**Table 1:** Demographic characteristics and clinical characteristics of RSV cases (*n* = 48).

Variable	RSV negative (*n* = 69)	RSV positive (*n* = 48)	*p*-value
**Demographics and clinical characteristics**			
Age group			
≤6 months	19 (27.54%)	24 (50.00%)	0.068
6 months–2 years	27 (39.13%)	16 (33.33%)	
2–5 years	16 (23.19%)	5 (10.42%)	
≥5 years	7 (10.14%)	3 (6.25%)	
Male	41 (59.42%)	33 (68.75%)	0.303
Duration of hospital stay (days)	8 (6–11)	10 (7–12)	0.256
**Symptoms and signs**			
Fever (temperature ≥ 37.3°C)	44 (63.77%)	30 (62.50%)	0.889
Cough	50 (72.46%)	40 (83.33%)	0.170
Sputum production	35 (50.72%)	34 (70.83)	0.030*
Wheezing	20 (28.99%)	29 (60.42%)	<0.01*
Nasal congestion	10 (14.49%)	12 (25.00%)	0.153
Rhinorrhea	11 (15.94%)	13 (27.08%)	0.142
Throat congestion	32 (46.38%)	24 (50.00%)	0.700
Convulsive seizures	21 (30.43%)	10 (20.83%)	0.247
Gastrointestinal symptoms	24 (34.78%)	14 (29.17%)	0.523
Rales	40 (57.97%)	39 (81.25%)	<0.01*
Respiratory failure	14 (20.29%)	14 (29.17%)	0.268
Abnormal respiratory rate, beats/min	49 (71.01%)	36 (75.00%)	0.634

Data are median (IQR), mean ± SD, or *n* (%). As appropriate, *p*-values were calculated by the *t*-test, Kruskal–Wallis test, χ^2^ test, or Fisher’s exact test. “∗” means *p* < 0.05.

**Table 2:** Laboratory, radiographic findings, treatments, and outcomes of 48 patients infected with RSV.

Variable	RSV negative (*n* = 69)	RSV positive (*n* = 48)	*p*-value
**Laboratory findings**			
Lymphocyte, × 10^9^ per L			
<2	21.00 (30.43%)	12.00 (25.00%)	0.831
2–7	43.00 (62.32%)	32.00 (66.67%)	
≥7	5.00 (7.25%)	4.00 (5.80%)	
Neutrophil, ×10^9^ per L	5.43 (2.64–9.38)	3.40 (1.89–7.03)	0.054
C reactive protein, mg/L (≥8)	30 (43.48%)	20 (41.76%)	0.846
Acidophil, ×10^9^ per L	0.02 (0.01–0.10)	0.03 (0.01–0.10)	0.931
Basophile, ×10^9^ per L	0.01 (0.00–0.02)	0.01 (0.01–0.02)	0.875
Monocyte, ×10^9^ per L	0.76 (0.49–1.19)	0.69 (0.53–0.98)	0.411
Erythrocyte, ×10^12^ per L	4.30 (3.73–4.57)	4.00 (3.51–4.38)	0.056
Hemoglobin	115 (107–126)	111 (104–120)	0.135
Neutrophils, ×10^9^ per L	303 (249–404)	336 (246–415)	0.372
Alanine aminotransferase, U/L	30 (19–41)	30 (18–40)	0.999
Aspartate aminotransferase, U/L	50 (35–65)	52 (43–63)	0.630
Creatinine, μmol/L	23 (17–29)	21 (17–25)	0.405
Creatine kinase, U/L	107 (67–179)	90 (68–152)	0.580
Creatine kinase isoenzyme, U/L	28 (20–38)	33 (25–45)	0.177
**Radiographic findings**			
Chest effusion	3/54 (5.56%)	2/34 (5.88%)	1.00
Chest shadow	6/56 (10.71%)	10/38 (26.32%)	0.048*
Abnormal respiratory sounds	25 (36.23%)	15 (31.25%)	0.576
**Treatments**			
Mechanical ventilation	9 (13.04%)	17 (35.42%)	0.004*
Antibiotics	43 (89.58%)	58 (84.06%)	0.392
**Disease severity status**			
Upper respiratory tract infection(URI)	32 (46.38%)	48 (41.03%)	0.246
NSCAP	19 (27.54%)	32 (27.35%)	
Bronchiolitis	10 (14.49%)	17 (14.53%)	
SCAP	8 (11.59%)	20 (17.09%)	
**Clinical outcomes**			
Positive	51 (73.91%)	37 (77.08%)	0.558
Ordinary	13 (18.84%)	11 (22.92%)	
Negative	5 (7.25%)	0 (0%)	

Data are median (IQR), mean ± SD, or *n* (%). As appropriate, *p*-values were calculated by the *t*-test, Kruskal–Wallis test, χ^2^ test, or Fisher’s exact test. “∗” means *p* < 0.05.

To avoid overfitting in the model, a multiple logistic regression analysis was performed to understand the association between using a ventilator and RSV infection. Based on the results of univariable results, six variables (age groups, gender, RSV infection, coinfections, comorbidity, and SCAP) were chosen for multivariable analysis (Table 3). RSV infection (OR = 9.55, 95% CI 1.87–48.64) was significantly higher in ventilator-used groups compared with non-ventilator-used groups in pediatric patients (*p* = 0·007). This indicates RSV infection is an independent risk factor for ventilator use. Furthermore, multiple logistic regression analysis showed that comorbidity was significantly associated with ventilator use (OR = 13.73, 95% CI 4.16–45.35), *p* < 0.001 (Table 3).

**Table 3:** Multivariable logistic regression of mechanical ventilation.

Factor	β	Wald	OR (95% CI)	*p*-value
Age group		2.10		0.553
≤6 months	−0.92	0.67	0.40 (0.04–3.62)	0.414
6 months–2 years	−0.73	1.11	0.48 (0.05–4.25)	0.510
2–5 years	0.19	0.03	1.20 (0.13–11.32)	0.872
≥5 years	Reference			
Gender				
Male	−0.18	0.10	0.84 (0.27–2.56)	0.753
Female	Reference			
RSV infection				
Yes	−0.18	7.38	9.55 (1.87–48.64)	0.007*
No	Reference			
Coinfections				
Yes	−1.31	2.11	0.27 (0.05–1.58)	0.146
No	Reference			
Comorbidity				
Yes	2.61	18.48	13.73 (4.16–45.35)	<0.001*
No	Reference			
SCAP				
Yes	0.84	1.31	2.31 (0.55–9.73)	0.252
No	Reference			

*p*-values were calculated by the logistic. “∗” means *p* < 0.05.

## 4 Discussion

Because implementation of public health measures to prevent the COVID-19 pandemic, the prevalence of various respiratory pathogens has been affected since 2020. In this study, RSV-infected cases were mainly found in hospitalized children (41.94%) in Bengbu, China, in winter 2021–2022. However, this result is different from that of studies in Beijing, China, in winter 2020–2021 (23). Our results showed that there were higher positive rates than several reports from different regions of China, such as Gansu in 2010–2019 (24), Beijing in 2015–2019 (23), and Suzhou in 2011–2014 (25). In Portugal, RSV positivity rates in children were up to approximately 60% between week 30 of 2021 and week 32 of 2021 and between week 39 and week 41 of 2021 (26). A study in England showed an unprecedented surge in respiratory syncytial virus activity in the summer of 2021, while RSV activity was lower than expected in winter 2021–2022 (27). However, the seasonal prevalence of RSV infection in this study still follows this pattern, where RSV infections primarily occur in the autumn and winter seasons, and the epidemic period of RSV infection is from November of the first year to February of the following year.

Previous studies show that prematurity and young age are independent risk factors for severe RSV infection (28). In this study, the majority (36.75%, 43/117) of the patients were younger than 6 months old, and the median age of RSV-positive patients was 1 year old (IQR: 0.3–2.5 years old), which was younger than that of RSV-negative patients (*p* < 0.01). These results were similar to previous reports (3, 29–31). By estimating the hospitalization burden of RSV-associated respiratory infections (RSV-RTI) in children under 5 years old in seven European countries, it was found that infants born in the first 2 months of the peak month of RSV infection had the highest hospitalization rate (32). The risk of RSV infection in young children was mainly associated with the high surface-area-to-volume ratio of the airway in young children’s development (33). This study indicated that RSV infection was the main cause of pneumonia, with RSV detected in 60% of SCAP cases, followed by 41.8% in bronchiolitis and 40.62% in NSCAP cases. However, an Italian study of children infected with RSV showed that the diagnosis was mainly bronchiolitis (34). Our results are also different from previous studies, which suggest that male individuals infected with RSV are more likely to develop severe illness than female patients (12, 35, 36). Interestingly, we found that the patients infected with RSV were more likely to experience clinical symptoms such as sputum, wheezing, and pulmonary rales, and require mechanical ventilation. Similar to our results, a multivariable regression in South Korea showed that increased odds of mechanical ventilation were associated with RSV infection (37). We still found that children infected with RSV-B required a higher proportion of mechanical ventilation compared with RSV-A, which is similar to the results of Hornsleth (38).

Similar to previous studies in China, German, Italian, and Kenyan (8, 39–43), ON1 and BA9 were the dominant strains in this study. The analysis of G gene variability found that the ON1 genotype was divided into two clusters (Figure 2A). In this study, all G gene sequences of clade 1 had four aa substitutions including T113I, V131D, N178G, and H258Q. Interestingly, the remaining 4 strains in ON1 formed clade 2, and these strains had common mutations: E224A, L247P, T282I, G296S, Y304H, S311P, L314P, and T320A. L247P was shown to be associated with immune escape (44). In addition, seven high-frequency amino acid replacements occurred in BA9. N178S mutation similar to ON1 was found in one BA9 strain, and the mutation in the central conserved region may lead to the emergence of new prevalent strains in future. Whether these mutations have an effect on the generation of new lineages still needs to be verified by subsequent surveillance.

The limitations of this study are the impact of the COVID-19 pandemic, the short surveillance time, and inconvenient follow-up. However, we still strictly adhere to the inclusion criteria of ARTI.

## 5 Conclusion

In summary, RSV was the number one pathogen in winter 2021–2022 among hospitalized children in Bengbu, China. RSV mainly occurs in children under 2 years old. ON1 of RSV-A and BA9 of RSV-B were the dominant genotypes in Bengbu in winter 2021–2022. These results indicate that long-term, continuous surveillance of RSV is necessary.

## Data availability statement

The datasets presented in this study can be found in online repositories. The names of the repository/repositories and accession number(s) can be found in the article/Supplementary material.

## Ethics statement

The studies involving humans were approved by Ethics Committee at the First Affiliated Hospital of Bengbu Medical College (BYYFY-2019KY38). The studies were conducted in accordance with the local legislation and institutional requirements. Written informed consent for participation in this study was provided by the participants’ legal guardians/next of kin. Written informed consent was obtained from the minor(s)’ legal guardian/next of kin for the publication of any potentially identifiable images or data included in this article.

## Author contributions

LH: Investigation, Software, Writing – original draft, Methodology, Resources, Validation. YX: Investigation, Writing – original draft, Writing – review & editing, Methodology, Resources. YY: Investigation, Methodology, Software, Writing – original draft, Resources, Formal analysis. HoD: Investigation, Methodology, Software, Writing – original draft. QL: Investigation, Software, Conceptualization, Methodology, Writing – original draft. ZC: Methodology, Software, Writing – original draft. HaD: Supervision, Writing – review & editing, Investigation. GM: Methodology, Supervision, Investigation, Writing – original draft. XW: Investigation, Software, Writing – original draft. YG: Investigation, Software, Writing – original draft. CZ: Investigation, Software, Supervision, Writing – review & editing, Conceptualization. JH: Formal analysis, Funding acquisition, Methodology, Resources, Supervision, Writing – review & editing. GL: Formal analysis, Investigation, Methodology, Resources, Supervision, Validation, Visualization, Writing – review & editing.

## Footnotes

^1^http://www.cbs.dtu.dk/services/NetNGlyc
