# Supplementary material for: Molecular epidemiology and clinical characteristics of respiratory syncytial virus in hospitalized children during winter 2021–2022 in Bengbu, China
Source: Front Public Health. 2024 Jan 3;11:1310293. doi: 10.3389/fpubh.2023.1310293 (PMC10791987; doi:10.3389/fpubh.2023.1310293)
Supplement: Supplementary file 1 [file Data_Sheet_1.pdf]

Table. S1 Demographic and clinical characteristics of RSV-A and RSV-B infections (n=45)

**Variable**	**RSV-A (n=34)**	**RSV-B (n=11)**	***P*-value**
**Demographics and clinical characteristics**			
Age group			
≤6 month yr	19 (55.88%)	4 (36.36%)	0.143
6month-2 yr	12 (35.29%)	3 (9.09%)	
2-5 yr	2 (5.88%)	2 (18.18%)	
≥5 yr	1 (2.94%)	2 (18.18%)	
Male	26 (76.47%)	5 (45. 45%)	0.071
Duration of hospital stay (days)	10 (7-12)	9 (6-12)	0.700
**Symptoms and signs**			
Fever (temperature ≥ 37.3 °C)	22 (64.71%)	7 (63.64%)	1.00
Cough	28 (82.35%)	10 (90.91%)	0.663
Sputum production	23 (67.65%)	10 (90.91%)	0.240
Wheezing	23 (67.65%)	5 (45.45%)	0.285

Nasal congestion	9 (26.47%)	3 (27.27%)	1.000
Rhinorrhea	9 (26.47%)	2 (18.18%)	0.705
Throat congestion	17 (50.00%)	5 (45.45%)	0.793
Convulsive seizures	6 (17.65%)	2 (18.18%)	1.000
Gastrointestinal symptoms	11 (32.35%)	2 (18.18%)	0.467
Rales	29 (85.29%)	9 (81.82%)	1.000
Respiratory failure	8 (23.53%)	5 (45.45%)	0.312

Data are median (IQR), mean±SD or n (%). As appropriate, p-values were calculated by the T-test, Kruskal–Wallis test, $\chi 2$ test, or Fisher's exact test. "*" means $p<0.05$.

Table. S2 Laboratory, radiographic findings, treatments and outcomes of patients infected with RSV-A and RSV-B

Variable	RSV-A (n=34)	RSV-B (n=11)	*P*-value
**Laboratory findings**			
Lymphocyte, × 10^9 per L			
<2	6 (17.65%)	4 (36.36%)	0.404
2-7	25 (73.53%)	6 (54.55%)	
≥7	3 (8.82%)	1 (9.09%)	
Neutrophil, ×10^9 per L	2.97 (1.84-4.96)	6.52 (2.02-8.43)	0.055
C reactive protein, mg/L(≥8)	10 (29.41%)	7 (63.64%)	0.072
Acidophil, ×10^9 per L	0.04(0.01-0.11)	0.02(0.01-0.04)	0.585
Basophile, ×10^9 per L	0.01(0.01-0.02)	0.01(0.00-0.02)	0.334
Monocyte, ×10^9 per L	0.66(0.51-0.94)	0.75(0.57-1.01)	0.731
Erythrocyte, ×10^{12}per L	3.94±0.61	3.75±0.94	0.447
Hemoglobin	110±12	107±29	0.765

Neutrophils, $\times 10^9$ per L	354±135	332±107	0.634
Alanine aminotransferase, U/L	29 (18-39)	36 (15-68)	0.362
Asparate aminotransferase, U/L	50 (42-60)	62 (45-67)	0.093
Creatinine, µmol/L	21 (18-26)	21 (16- 22)	0.781
Creatine kinase, U/L	90 (70-148)	98 (58-236)	0.885
Creatine kinase isoenzyme, U/L	39±16	28±16	0.069
**Radiographic findings**			
Chest effusion	1/24 (4.17%)	1/7 (14.29%)	0.406
Chest shadow	5/26 (19.23%)	5/9 (55.56%)	0.081
**Treatments**			
Mechanical ventilation	9 (26.47%)	7 (63.64%)	0.035*
Antibiotics	31 (91.18%)	3 (27.27%)	<0.001*
**Disease severity status**			
URTI	11 (32.35%)	3 (27.27%)	0.380

Pneumonia	11 (32.35%)	2 (18.18%)	
Bronchiolitis	6 (17.65%)	1 (9.09%)	
Severe pneumonia	6 (17.65%)	5 (45.45%)	
**Clinical outcomes**			
Positive	27 (79.41%)	7 (63.64%)	0.304
Ordinary	7 (20.59%)	4 (36.36%)	
Negative	0 (0%)	0 (0%)	

Data are median (IQR), mean±SD or n (%). As appropriate, p-values were calculated by the T-test, Kruskal–Wallis test, χ2 test, or Fisher's exact test. "*" means $p<0.05$.
