# Supplementary figures and images for: Molecular epidemiology and clinical characteristics of respiratory syncytial virus in hospitalized children during winter 2021–2022 in Bengbu, China
Source: Front Public Health. 2024 Jan 3;11:1310293. doi: 10.3389/fpubh.2023.1310293 (PMC10791987; doi:10.3389/fpubh.2023.1310293)

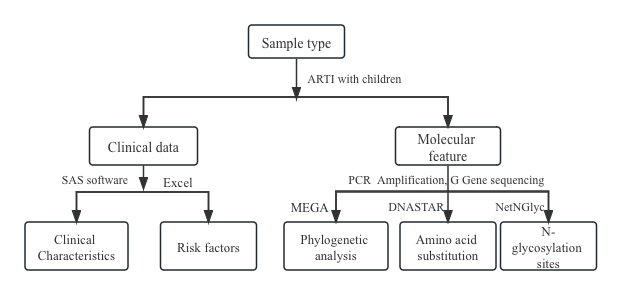

Supplement: Supplementary file 2 [file Image_1.jpeg]
